# Supplementary material for: Effects of trading networks on the risk of bovine tuberculosis incidents on cattle farms in Great Britain
Source: R Soc Open Sci. 2020 Apr 22;7(4):191806. doi: 10.1098/rsos.191806 (PMC7211880; doi:10.1098/rsos.191806)
Supplement: Contact chains and bTB supplement [file rsos191806supp1.pdf]

Effects of trading networks on risks of bovine tuberculosis infection on cattle farms in Great Britain

Helen R. Fielding, Trevelyan J. McKinley, Richard J. Delahay, Matthew J. Silk, and Robbie A. McDonald*

*Corresponding author. *Email:* r.mcdonald@exeter.ac.uk

Supplementary Materials

Methods

Regional definitions: England risk areas and Wales

During the study period (2010–2016) there were multiple changes to bovine tuberculosis (bTB) surveillance policies in Great Britain. Until the end of 2012, herds in England were tested every 1, 2, 3 or 4 years, according to the classification of their parish (smaller geographical areas within counties). On 1st January 2013, three risk areas were implemented in England: the England High Risk Area, where infection is considered endemic in badgers and cattle, the England Low Risk Area, where there is low incidence, and the England Edge area, which encompassed regions deemed to be at highest risk of endemicity in the near future [1] (see Fig. S1 for map). In most cases, whole counties lie within a single risk area, however five counties (East Sussex, Oxfordshire, Warwickshire, Derbyshire and Cheshire) were divided between the England High Risk Area and the England Edge Area along parish boundaries. For the purposes of this study, we defined these risk areas at the parish-level using reference data obtained from the Animal and Plant Health Agency (APHA) and as per the 2015 surveillance report by the APHA [2]. After the risk areas were implemented (2013 onwards), herds in the England High Risk Area and England Edge Area were tested at least annually while herds in the England Low Risk Area and Scotland were tested every four years, although in Scotland some farms were exempt from live animal testing according to risk-based analyses [3]. Despite marked regional differences in herd incidence (and devolved bTB policy) within Wales, we elected to categorise the entire country as one risk area, since during the study period herds in all Welsh regions were tested annually.

Herd type definition

Using Cattle Tracing System (CTS) data, *suckler* farms were defined by a majority of female beef animals, aiming to capture herds where calves are reared by their dams (cow-calf systems). *Dairy* farms were defined by a majority of female dairy cattle, identifying herds producing milk commercially. *Fattening* units were defined by a male animal majority, identifying herds that mainly

reared cattle for beef production. *Mixed* farms were those where no one breed type or sex constituted more than 50% of the herd.

Constructing networks

In constructing our networks, only farm-to-farm movements were analysed, omitting premises where animals resided for less than one day. Movements between farms via transitory locations, such as markets, were classed as a direct link with the transitory location excluded, as we considered short-term locations of minimal importance to the transmission of bTB [4], in contrast to their importance in the spread of highly infectious diseases. Multiple animals moving to and from the same farms on the same day were aggregated to form a single directed connection.

Constructing contact chains

The 'root farm' is at the start of the chain. Any farms that have directly sold animals to the root farm in the previous year are considered to be at level one in the contact chain. Farms initiating *previous* movements *onto* level one farms (i.e. those made before the move to the root farm) are considered to be at the second level away from the root farm (i.e. two movements in a temporal sequence connect the root farm and a farm at the second level of the chain), farms initiating movements of cattle onto second level farms are considered to be at the third level, and so on. All movements creating connections up the chain precede the movement between the lower levels and thus maintain a possible infection pathway. The resulting chain is fully-connected by animals that have been on the same farm for at least one day and therefore have the potential, in principle, to transmit infection between farms. Any farm in the ICC of a root farm is considered a 'source farm'. We generated twenty-four ICCs for each active root farm using movements recorded during the study period. The twenty-four chains end sequentially one month apart and each include the previous 12 months of movements. We chose 12-month periods as a conservative estimate of how long a bTB infected farm might trade without being detected. We calculated the *number* of farms in each ICC (source farms) but encountered computational limitations in *identifying* farms at more than eight levels away in extremely large chains, and so each chain was curtailed at this point. We estimate approximately 28% of root farms had source farms above level 8, and these source farms were not included in the analysis. However, given that the likely effect of farms diminishes at greater distances in the ICC and that farmers have less and less control over connections as the chain builds, we consider this is sufficient to capture the more important risk factors related to bTB transmission.

Selecting contact chain variables

Using the method described in the multivariable analysis section in Methods, we split the contact chain variables into two groups based on whether we expected them to increase or decrease the odds of bTB on the root farm in the univariate analysis (Fig. S3). First, we ran the baseline model with each cumulative variable from both groups. We selected the variable from each group that was included the model with the lowest AIC. For the increased risk group this was the number of source farms in the England High Risk Area at levels 1–3 and for the protective group this was the number of source farms in Scotland at levels 1–8 (Fig. S3). Second, to determine if the selected variables had an impact on the AICs of one another, we ran a sensitivity analysis in which we repeated the previous steps by including each variable from each group (e.g. the protective group: number of farms in the England Low Risk Area at levels 1, 1–2, 1–3, etc. up to levels 1–8 and the number of farms in Scotland at levels 1, 1–2, 1–3, etc. up to levels 1–8) with the baseline model, but also added the variable from the other group that was selected in the first stage (e.g. selected variable from the increased risk group: the number of farms at levels 1–3 in the England high risk area). Initially, the variable in the model with the lowest AIC was the number of Scottish farms at levels 1–8 (Fig. S3, however, after adding in the 'best' variable from the increased risk group, and testing all variables from the protective group, the model with the lowest AIC became the number of farms at levels 1–8 in the Low Risk Area (Fig. S3). The best performing representative variable from the increased risk group (number of farms at levels 1–3 in the England high risk area) consistently had the lowest AICs with the either the number of farms in the England Low Risk Area or Scottish farms at levels 1–8 (Fig. S3). As the model containing both the number of farms in the England High Risk Area at levels 1–3 and the number of farms in the England Low Risk Area at levels 1–8, gave the lowest AIC, we selected the England Low Risk Area variable from the protective group. We also performed the above sensitivity analysis with and without the local risk factor (the proportion of farms with a bTB incident in 2010–2014 at a radius of 0–8km from the root farm). AIC values were consistently higher, by a mean of 3.22% (standard deviation = 0.05%), when the local risk factor was not included, therefore it was included in the final model (Fig. S3).

References

1. Lawes JR, Harris KA, Brouwer A, Broughan JM, Smith NH, Upton PA. 2016 Bovine TB surveillance in Great Britain in 2014. *Vet. Rec.* **178**, 310–315. (doi:10.1136/vr.i1616)
2. Animal and Plant Health Agency. 2016 *Bovine tuberculosis in Great Britain - Surveillance data for 2015 & historical trends.* Animal and Plant Health Agency, Addlestone, UK.
3. The Scottish Government. 2011 *Risk-based surveillance for tuberculosis in cattle (bTB). Report No. UGW/003/10.* The Scottish Government, Edinburgh UK.

4. Skuce R, Allen AR, Mcdowell SWJ. 2011 *Bovine tuberculosis (TB): A review of cattle-to-cattle transmission, risk factors and susceptibility.* Agri-food and Biosciences Institute, Belfast, UK.

Supplementary figures

Figure S1. Map of Great Britain illustrating the regional areas used in this study: Scotland, Wales, England High Risk Area, England Low Risk Area and England Edge Area. Bovine tuberculosis risk areas of England were defined at parish level for our analyses, as defined by the Animal and Plant Health Agency bovine tuberculosis surveillance report 2015 [2]. Counties that were partly in the England High Risk Area and partly in the England Edge Area from 2013 to the study end (Cheshire, Derbyshire, Warwickshire, Oxfordshire, East Sussex) are coloured on the map as the England High Risk Area and denoted with a white asterisk.

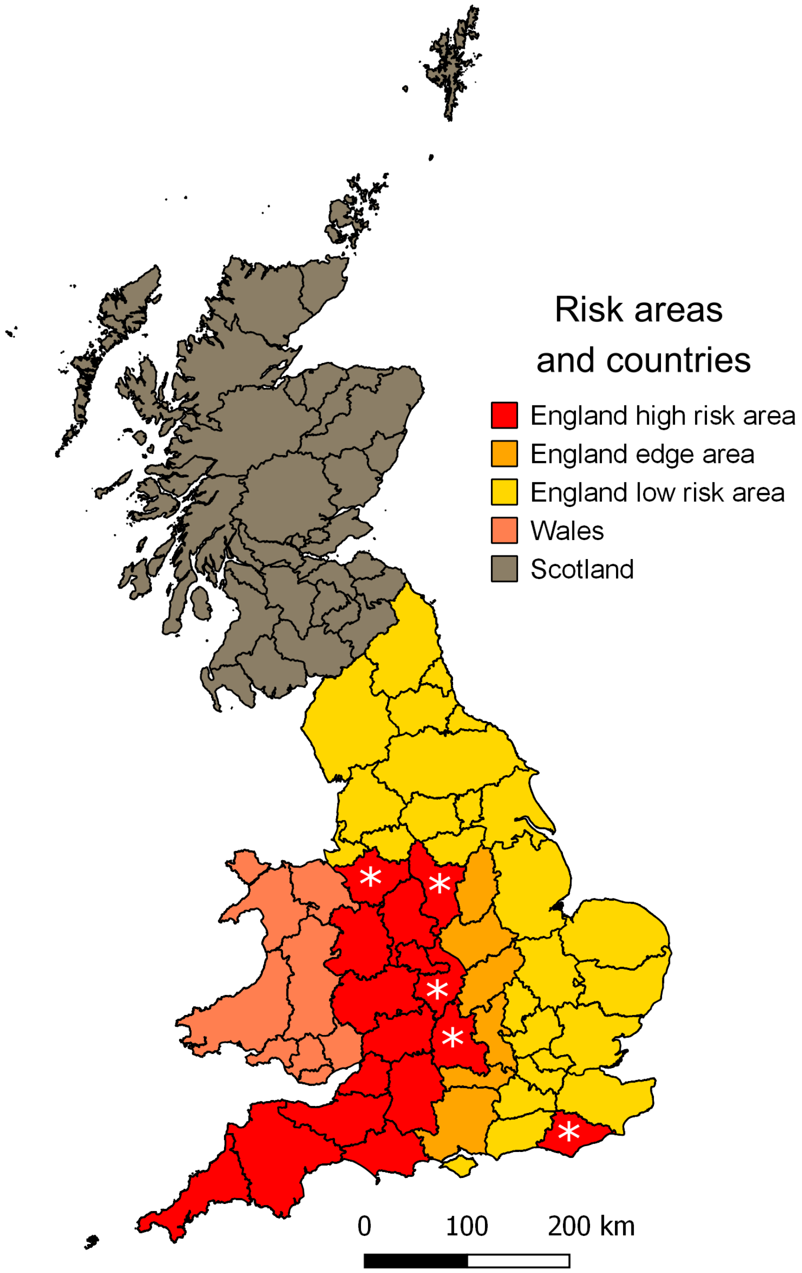

Figure S2. Relative performance of test models to inform variable selection for inclusion in the final multivariable model. Variables tested are for the proportion of farms with a bTB incident 2010–2014 at increasing distance from the root farm. AIC values are shown for the baseline model, without any local bTB variable, and the five competing multivariable logistic regression models which included the proportion of farms within 0–2 km, 0–4 km, 0–6 km, 0–8 km, and 0–10 km of the root farm respectively. All models used the response variable of whether the root farm had a bTB incident 2015-2016 and all included the baseline explanatory variables: root farm risk area, root herd size, root herd type, whether or not root farm had bTB incident 2010-2014, root farm betweenness, the mean number of source farms in the ingoing contact chain (ICC), whether or not the farm purchased cattle, the mean number of cattle purchased annually, whether or not the farm purchased any animals from the England High Risk Area. The test model that included the proportion of farms within 8 km of the root farm with a bTB incident 2010–2014 had the lowest AIC value and was therefore included in our final multivariable model.

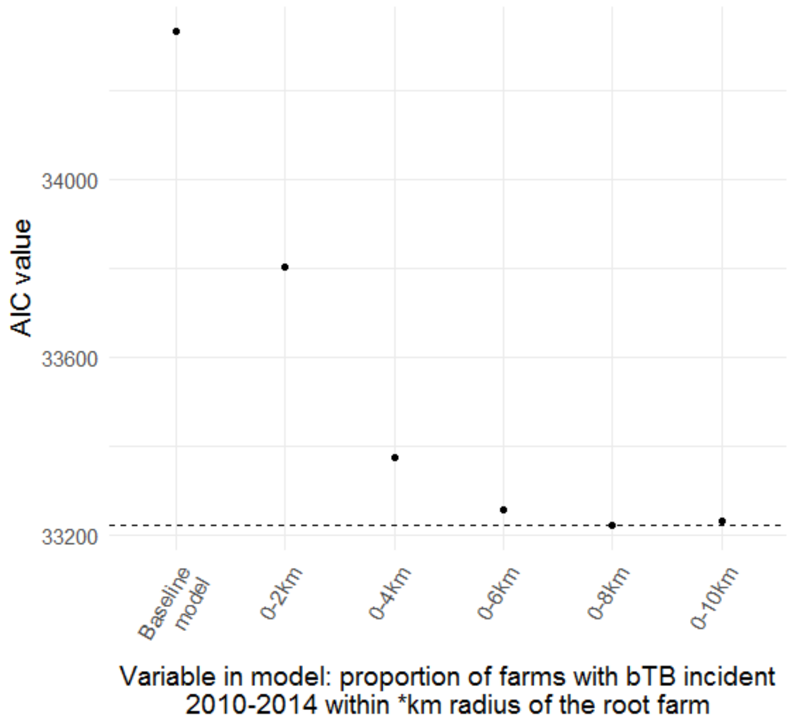

Figure S3. Relative performance of test models to inform variable selection for inclusion in the final GB multivariable model. Variable selection was based on the combination of parameters included in the model with the lowest AIC value. Each variable within a category (e.g. increased risk) was tested with the selected variable from the other category (e.g. decreased risk) and the baseline model. Additionally, all variables (from left to right on the x-axis) were run with (bottom row) and without (top row) the local bTB incident variable (0–8 km see Fig. S2) and models always performed better with this variable included. The optimum combination of variables defined by lowest AIC values included the number of farms in the England High Risk Area at levels 1-3 in the ICC (grey line with circles, bottom left panel) and the number of farms in the England Low Risk Area at levels 1-8 in the ICC (orange line with triangles, bottom right panel). All models include the baseline explanatory variables root farm risk area, herd size, herd type, bTB incident 2010-2014 (yes/no), betweenness, the mean number of source farms in the ingoing contact chain (ICC), purchase of cattle (yes/no), the mean number of cattle purchased annually, and purchase of any animals from the England High Risk Area (yes/no).

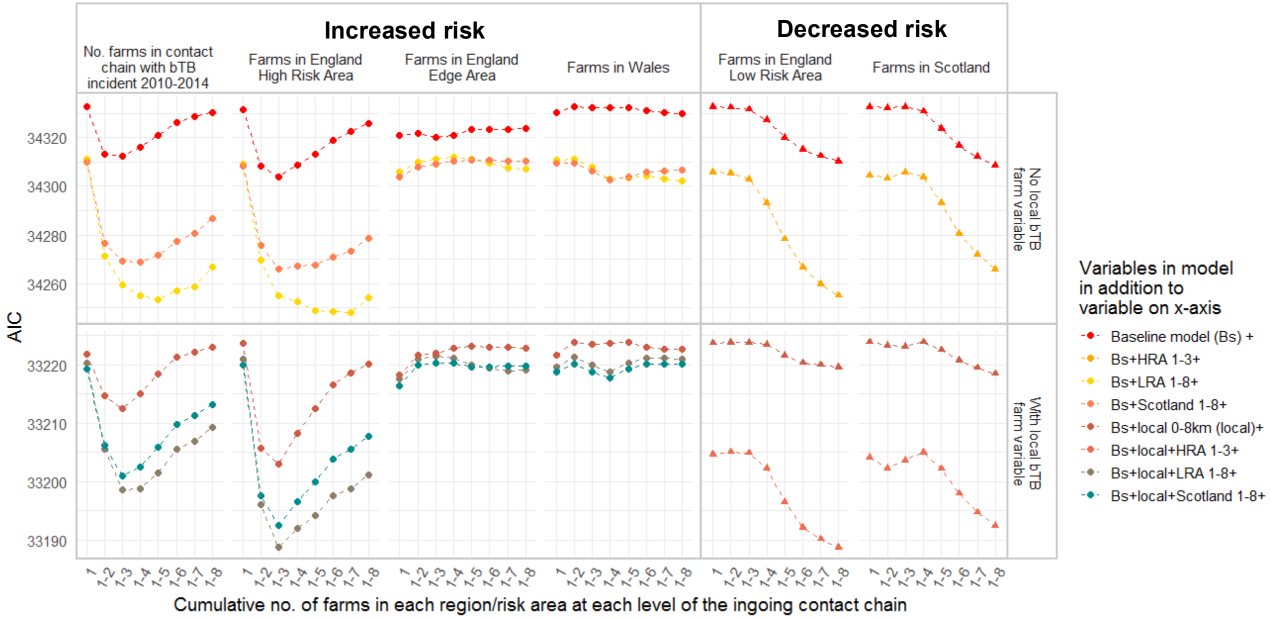

Figure S4. Performance of final GB and regional multivariable models in predicting bTB incidents on root farms 2015–2016. Plots show model a) sensitivity, b) specificity, c) area under receiver operating characteristic curve, d) positive predictive value, e) negative predictive value, and f) accuracy. Models include the explanatory variables: root herd size, bTB incident 2010–2014, herd type, betweenness, cattle purchased (yes/no), the number of source farms in the ICC, the proportion of farms within 8km of the root farm with bTB incident 2010–2014, cattle purchased from England High Risk Area (yes/no), number of farms in England High Risk Area at levels 1–3 of the contact chain, number of farms in the England Low Risk Area at levels 1–8 of the contact chain, and root farm region (GB model only). We were unable to calculate a positive predictive value for the Scottish model as no bTB incidents were predicted to occur.

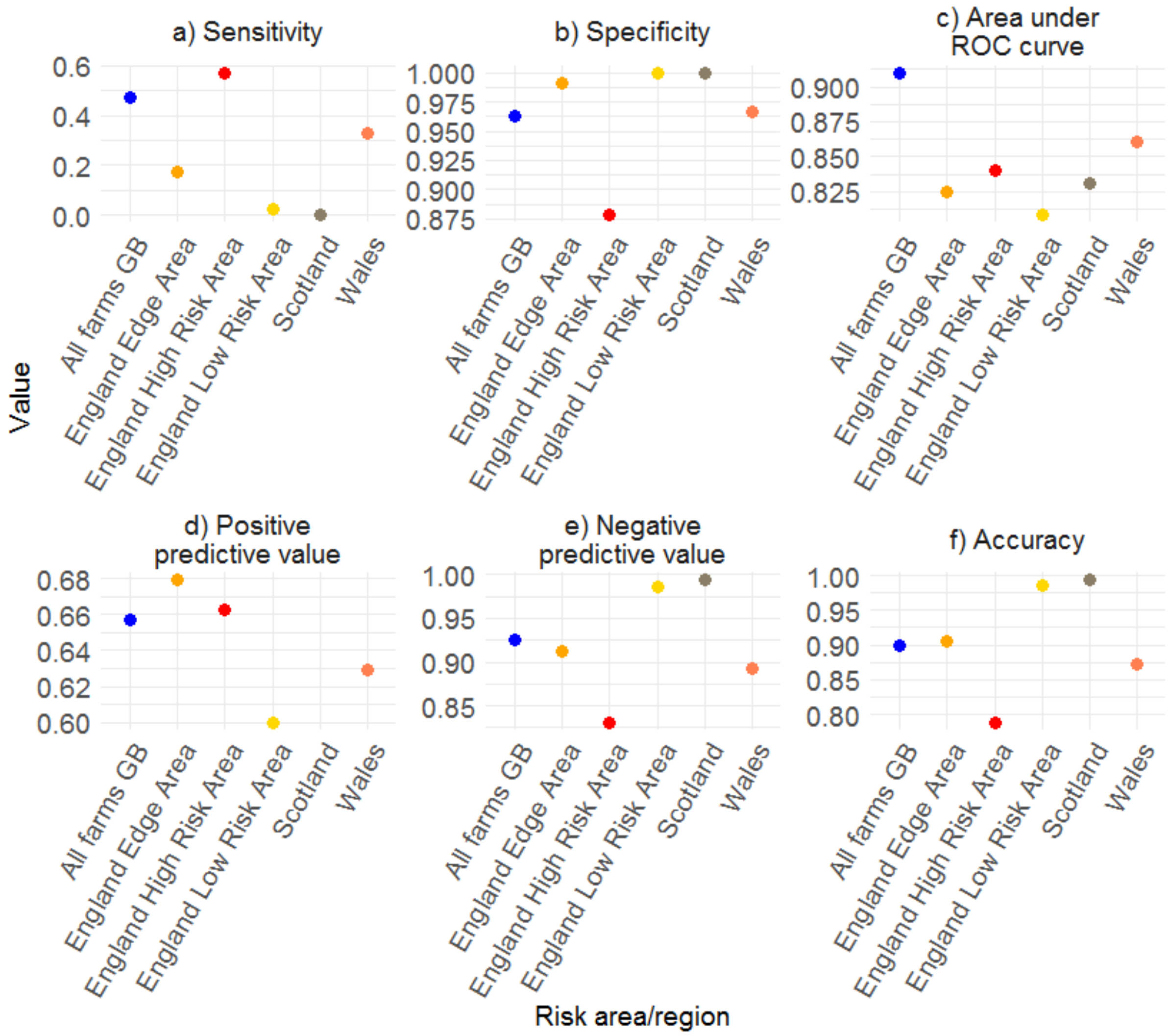

Figure S5. Mean distance (km) to the root farm from source farms at each level of the ingoing contact chain in a trading network of cattle farms in Great Britain 2012 to 2014. The box plots indicate the median and 25th and 75th percentiles, the upper and lower whiskers extend to the largest or smallest value no further than 1.5 times the interquartile range and data beyond this range are plotted as outlying points.

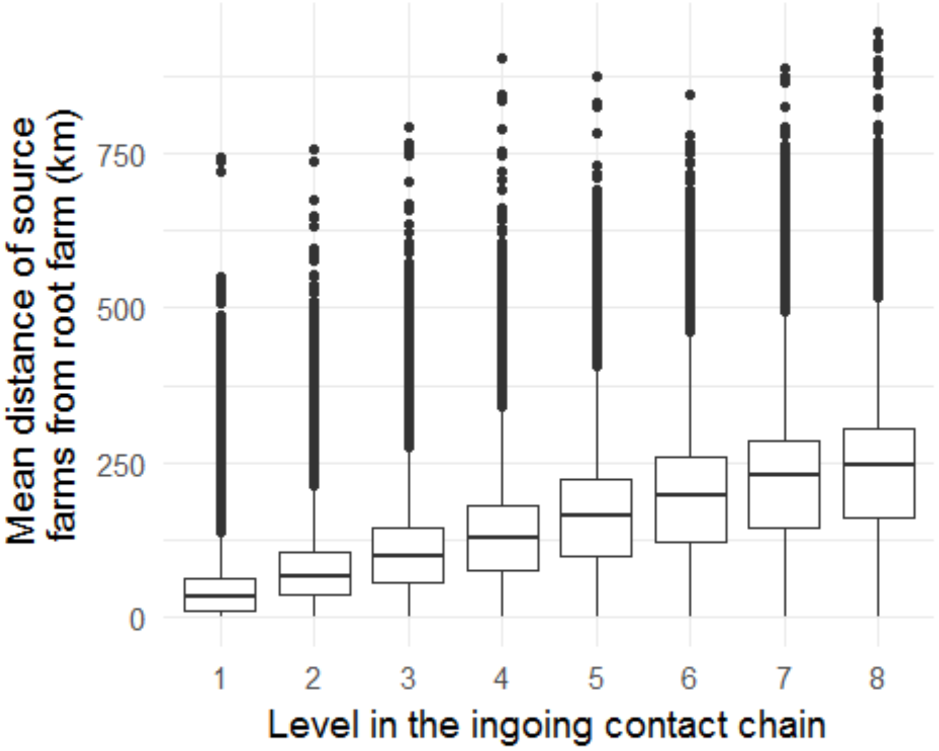

Table S1. Relative numbers of study farms with bovine tuberculosis (bTB) incidents during 2015–2016. Incidents are classified as Officially Tuberculosis Free suspended (OTF-S) or withdrawn (OTF-W) and numbers of study farms with bTB incidents 2015–2016 that also had a bTB incident 2010–2014 in different regions and Great Britain as a whole are shown. Percentages shown are of those farms that experienced a bTB incident, not all study farms. *Five farms had unclassified bTB incidents, 4 in the England High Risk Area and 1 in the England Edge Area.

	Region					Great Britain
	England High Risk Area	England Edge Area	England Low Risk Area	Wales	Scotland	
Number of root farms with OTF-S bTB incident 2015–2016	1234 (19.1%)	304 (42.3%)	181 (63.5%)	210 (12.4%)	53 (72.6%)	1982 (21.5%)
Number of root farms with OTF-W bTB incident 2015–2016	5213 (80.8%)	413 (57.5%)	104 (36.5%)	1486 (87.6%)	20 (27.4%)	7236 (78.5%)
Number of root farms with bTB incident (OTF-S or W) during 2015–2016 *and* during 2010–2014	5092 (78.9%)	322 (44.8%)	60 (21.1%)	1229 (72.5%)	21 (28.8%)	6724 (72.9%)
Total number of root farms with bTB incident (OTF-S or W) 2015–2016	6451*	718*	285	1696	73	9223

Table S2. Effect sizes of explanatory variables on the odds of a bTB incident on the root farm in 2015–2016. Odds ratios with 95% confidence intervals are from our multivariable logistic regression analysis using regional data from the England High Risk Area, the England Edge Area, the England Low Risk Area, Wales, and Scotland. Odds ratios of continuous variables are standardised as the odds associated with the difference between the 10th and 90th percentiles of the raw data.

Region	Parameter		10th percentile (raw data)	90th percentile (raw data)	Odds ratio	2.5% confidence limit	97.5% confidence limit
England High Risk Area (n = 21892)	Root farm herd type	Mixed			Baseline		
		Dairy	-	-	1.34	1.17	1.55
		Fat	-	-	0.89	0.77	1.04
		Suckler	-	-	1.09	0.97	1.24
	Root farm bTB 2010-2014		-	-	2.75	2.54	2.98
	Cattle purchased by root farm		-	-	0.97	0.83	1.13
	Mean number of farms in ICC	1st quartile (0–1)			Baseline		
		2nd quartile (2–421)	-	-	1.06	0.93	1.20
		3rd quartile (422–5601)	-	-	1.15	1.01	1.32
		4th quartile (5602–39676)	-	-	1.25	1.02	1.54
	Cattle purchased direct from England High Risk Area		-	-	1.21	1.00	1.47
	Root farm herd size		4	291	22.34	19.00	26.31
	Mean number of purchased cattle		0	201	1.00	0.97	1.03
	Root farm betweenness		0	277523	0.99	0.99	1.00
	Proportion of farms within 8km with bTB 2010-2014		0.26	0.59	3.03	2.74	3.35
	No. farms in England High Risk Area at levels 1-3		0	1181	1.21	1.11	1.33
	No. farms in England Low Risk Area at levels 1-8		0	8581	0.71	0.58	0.87
England Edge Area (n = 6960)	Root farm herd type	Mixed			Baseline		
		Dairy	-	-	1.46	1.03	2.10
		Fat	-	-	1.00	0.69	1.47
		Suckler	-	-	1.30	0.95	1.81
	Root farm bTB 2010-2014		-	-	2.33	1.91	2.82
	Cattle purchased by root farm		-	-	0.89	0.69	1.16
	Mean number of farms in ICC	1st quartile (0–1)			Baseline		
		2nd quartile (2–344)	-	-	1.20	0.87	1.67
		3rd quartile (345–6204)	-	-	1.35	0.93	1.95
		4th quartile (6205–37934)	-	-	1.19	0.66	2.12
	Cattle purchased direct from England High Risk Area		-	-	1.24	1.00	1.54
	Root farm herd size		3	257	16.23	11.11	23.96
	Mean number of purchased cattle		0	241	1.01	0.96	1.07
	Root farm betweenness		0	349309	1.00	0.99	1.00
	Proportion of farms within 8km with bTB 2010-2014		0.04	0.25	3.16	3	3.81
	No. farms in England High Risk Area at levels 1-3		0	900	1.15	0.99	1.35
	No. farms in England Low Risk Area at levels 1-8		0	10715	0.76	0.43	1.36

Region	Parameter		10th percentile (raw data)	90th percentile (raw data)	Odds ratio	2.5% confidence limit	97.5% confidence limit
England Low Risk Area (n = 18942)	Root farm herd type	Mixed			Baseline		
		Dairy	-	-	0.86	0.54	1.41
		Fat	-	-	0.81	0.49	1.36
		Suckler	-	-	0.80	0.53	1.24
	Root farm bTB 2010-2014		-	-	4.57	3.25	6.35
	Cattle purchased by root farm		-	-	1.38	0.92	2.11
	Mean number of farms in ICC	1st quartile (0–2)			Baseline		
		2nd quartile (3–1522)	-	-	1.03	0.65	1.68
		3rd quartile (1523–8464)	-	-	1.42	0.84	2.44
		4th quartile (8465–39185)	-	-	2.54	1.10	5.79
	Cattle purchased direct from England High Risk Area		-	-	1.48	1.06	2.04
	Root farm herd size		3	255	14.29	8.52	24.38
	Mean number of purchased cattle		0	225	1.01	0.97	1.04
	Root farm betweenness		0	395549	1.00	0.99	1.00
	Proportion of farms within 8km with bTB 2010-2014		0	0.06	1.64	1.34	1.98
	No. farms in England High Risk Area at levels 1-3		0	494	1.18	1.08	1.28
	No. farms in England Low Risk Area at levels 1-8		0	11932	0.21	0.09	0.49
Wales (n = 11525)	Root farm herd type	Mixed			Baseline		
		Dairy	-	-	1.31	0.99	1.75
		Fat	-	-	0.97	0.70	1.35
		Suckler	-	-	1.00	0.77	1.31
	Root farm bTB 2010-2014		-	-	2.60	2.27	2.99
	Cattle purchased by root farm		-	-	1.04	0.86	1.25
	Mean number of farms in ICC	1st quartile (0–1)			Baseline		
		2nd quartile (2–459)	-	-	0.92	0.74	1.15
		3rd quartile (460–4404)	-	-	0.95	0.75	1.20
		4th quartile (4405–33893)	-	-	0.86	0.60	1.24
	Cattle purchased direct from England High Risk Area		-	-	1.15	0.99	1.33
	Root farm herd size		5	219	14.65	11.12	19.39
	Mean number of purchased cattle		0	127	1.02	0.97	1.07
	Root farm betweenness		0	225091	1.00	0.99	1.01
	Proportion of farms within 8km with bTB 2010-2014		0.06	0.54	7.95	6.52	9.73
	No. farms in England High Risk Area at levels 1-3		0	317	1.04	0.95	1.13
	No. farms in England Low Risk Area at levels 1-8		0	7065	0.95	0.68	1.31

Region	Parameter		10th percentile (raw data)	90th percentile (raw data)	Odds ratio	2.5% confidence limit	97.5% confidence limit
Scotland (n = 11777)	Root farm herd type	Mixed			Baseline		
		Dairy	-	-	0.53	0.16	2.13
		Fat	-	-	0.72	0.18	3.09
		Suckler	-	-	0.99	0.39	3.35
	Root farm bTB 2010-2014		-	-	16.26	9.06	28.38
	Cattle purchased by root farm		-	-	1.20	0.46	3.55
	Mean number of farms in ICC	1st quartile (0–1)			Baseline		
		2nd quartile (2–632)	-	-	0.98	0.37	3.10
		3rd quartile (633–6682)	-	-	1.15	0.39	3.90
		4th quartile (6683–36712)	-	-	1.58	0.31	8.33
	Cattle purchased direct from England High Risk Area		-	-	0.46	0.09	1.46
	Root farm herd size		4	364	13.64	4.69	42.68
	Mean number of purchased cattle		0	198	0.98	0.90	1.03
	Root farm betweenness		0	286021	1.00	0.99	1.00
	Proportion of farms within 8km with bTB 2010-2014		0	0.05	1.14	0.80	1.41
	No. farms in England High Risk Area at levels 1-3		0	21	1.04	1.02	1.05
	No. farms in England Low Risk Area at levels 1-8		0	10386	0.69	0.16	2.96
